# Association between preoperative albumin and postoperative pneumonia in patients undergoing major non-cardiac surgery

**DOI:** 10.3389/fnut.2025.1642756

**Published:** 2025-10-23

**Authors:** Qian-Yun Pang, Yu-Mei Feng, Ya-Jun Yang, Hong-Liang Liu

**Affiliations:** ^1^Department of Anesthesiology, Chongqing University Cancer Hospital, Chongqing, China; ^2^Department of Anesthesiology, Zhuhai City People’s Hospital (The Affiliated Hospital of Beijing Institute of Technology, Zhuhai Clinical Medical College of Jinan University), Zhuhai, Guangdong, China

**Keywords:** postoperative pneumonia, albumin, non-cardiac surgery, dose–response, restricted cubic spline

## Abstract

**Background:**

Postoperative pneumonia is a common and serious complication following non-cardiac surgery. However, the relationship between preoperative albumin levels and postoperative pneumonia remains unclear in major non-cardiac surgeries, and the cut-off value for predicting postoperative pneumonia has yet to be determined.

**Methods:**

Data from patients who underwent non-cardiac surgery between January 1, 2019, and December 31, 2022, were extracted from the institutional electronic medical records of Chongqing University Cancer Hospital. The primary exposure of interest was preoperative albumin levels, and the outcome was postoperative in-hospital pneumonia.

**Results:**

The results revealed a non-linear dose–response relationship between preoperative albumin levels and postoperative pneumonia. A 1 g/L increase in preoperative albumin levels was associated with a 4.4% reduction in adjusted odds ratio (aOR) of postoperative pneumonia (aOR: 0.956, 95% CI: 0.940–0.973, *p* < 0.001). Patients with preoperative albumin levels <38.9 g/L or between 38.9 and 45.3 g/L had a 67.4 and 30.3% higher OR, respectively, compared to those with levels >45.3 g/L (aOR: 1.674, 95% CI: 1.313–2.135, *p* < 0.001; and aOR: 1.303, 95% CI: 1.045–1.624, *p* = 0.019). Subgroup analyses indicated that the risk of developing pneumonia was more pronounced in younger, female, and healthier patients.

**Conclusion:**

These findings suggest that preoperative albumin levels are significantly associated with the development of postoperative pneumonia in patients undergoing major non-cardiac surgery. A preoperative albumin level of <45.3 g/L may serve as a predictive marker for postoperative pneumonia.

## 1 Introduction

Postoperative pneumonia is a common and serious complication, with an incidence of 2–3% in non-cardiac surgery (1). It can lead to increased ICU admissions and a higher risk of postoperative morbidity and mortality (2, 3). Studies have shown that postoperative pneumonia can prolong hospitalization by 6–9 days (4), significantly increasing hospitalization costs. Therefore, identifying predictors of postoperative pneumonia may help reduce its incidence.

Albumin is the most abundant protein in human plasma, and a level below 35 g/L is defined as hypoalbuminemia (5). Hypoalbuminemia often develops before surgery due to malnutrition, malignancy, diabetes, and other conditions. Preoperative hypoalbuminemia is an independent risk factor for postoperative complications in lower extremity surgeries (6–10). However, evidence regarding the relationship between preoperative albumin levels and postoperative pneumonia in major non-cardiac surgeries remains limited. To our knowledge, the optimal preoperative albumin level for predicting postoperative pneumonia and whether the clinical threshold of 35 g/L is sufficiently stringent remain unclear.

In this retrospective study, we analyzed data from non-cardiac surgical patients to determine the association between preoperative albumin levels and postoperative in-hospital pneumonia. Additionally, we aimed to identify the cut-off value of preoperative albumin that could predict postoperative in-hospital pneumonia.

## 2 Methods

### 2.1 Study design

This retrospective study was approved by the Institutional Ethics Committee of Chongqing University Cancer Hospital, China (approval number: CZLS2024020-A). Due to the anonymized nature of the data and the retrospective design of the study, the requirement for informed consent from patients was waived by the Institutional Ethics Committee of Chongqing University Cancer Hospital.

### 2.2 Patients

Patients who underwent major non-cardiac surgery between January 1, 2019, and December 31, 2022, were initially enrolled. The inclusion criteria were: age ≥18 years, elective major non-cardiac surgery, surgical duration ≥2 h, and general anesthesia with intubation. The exclusion criteria were: patients with pneumonia or systemic infectious diseases within 1 month before surgery, and those with missing data on preoperative albumin levels or postoperative pneumonia diagnosis.

### 2.3 Data collection

The institutional electronic medical records of Chongqing University Cancer Hospital were reviewed from January 1, 2019, to December 31, 2022, using a validated automated data search software. Data from surgical patients who met the inclusion criteria were extracted based on clinical relevance and findings from previous studies. All data were collected using a self-designed case report form. The following variables were extracted: patient characteristics [age, sex, body mass index (BMI), ASA classification, smoking status]; and comorbidities including heart disease, hypertension, diabetes, renal disease, liver disease, cancer, stroke, chronic obstructive pulmonary disease (COPD), asthma, thyroid disease. Preoperative laboratory test results: hemoglobin (Hb), fasting glucose, white blood cell count (WBC), and albumin levels. Intraoperative variables: body temperature, mean arterial pressure (MAP), fluid infusion rate, and surgical duration.

### 2.4 Preoperative albumin measurement

The primary exposure of interest was the preoperative albumin level, which was measured at the central laboratory of Chongqing University Cancer Hospital after hospital admission but before surgery. If multiple measurements were taken before surgery, the result closest to the surgery date was used for analysis. Two definitions of cutoff points for albumin were evaluated: Clinical cutoff points: <35 g/L and ≥35 g/L. Cutoff points based on the first and third tertiles of albumin levels from the restricted cubic spline model.

### 2.5 Outcome measurement

The outcome of interest was postoperative in-hospital pneumonia. Cases were initially identified based on the diagnosis of postoperative pneumonia and subsequently reviewed to confirm the diagnosis according to the criteria established by the Centers for Disease Control and Prevention of the United States (CDC, 2008) (11).

### 2.6 Statistical analysis

Continuous variables were presented as median and interquartile range (IQR), while categorical variables were presented as frequencies and percentages [*n* (%)]. A restricted cubic spline model was used to evaluate the dose–response relationship between preoperative albumin levels and postoperative pneumonia. Preoperative albumin levels were analyzed as both continuous and categorized variables based on clinical criteria (<35 g/L and ≥35 g/L) and the first and third tertiles of preoperative albumin levels. Patients with missing data on preoperative albumin levels or postoperative pneumonia diagnosis were excluded from the final analysis. For other variables with missing values (<5% of data), the missing values were imputed using the median of each cohort, and all data were included in subsequent multivariable analyses.

Odds ratios (OR) and 95% confidence intervals (95% CI) for the risk of postoperative pneumonia were calculated using logistic regression. Both unadjusted and adjusted models (adjusted for all variables listed in Table 1) were analyzed. Subgroup analyses were conducted by stratifying the population based on age (<65 years, ≥65 years), sex (male, female), ASA classification (<III, ≥III), smoking status (present, absent), and surgical duration (<3 h, ≥3 h). Sensitivity analysis was conducted after excluding patients with hemoglobin <100 g/L.

**Table 1:** The clinical characteristics of patients undergoing non-cardiac surgery.

Variable	All patients (*n* = 28,044)	AL ≥ 45.3 g/L (*n* = 7,044)	38.9 g/L ≤ ALB<45.3 g/L (*n* = 14,069)	ALB < 38.9 g/L (*n* = 6,931)
Preoperative ALB (g/L)	42.1 (38.9, 45.4)	47.5 (46.3, 49.2%)	42.1 (40.6, 43.7)	36.6 (34.6, 37.9)
Age (year)	52 (45, 62)	49 (41, 56)	52 (46, 62)	57 (49, 67)
Gender				
Male	9,277 (33.08%)	1,737 (24.66%)	4,537 (32.19)	3,003 (43.33%)
Female	18,767 (66.92%)	5,307 (75.34%)	9,532 (67.62%)	3,928 (56.67%)
BMI	23.4 (21.4, 25.7)	23.4 (21.4, 25.6)	23.7 (21.7, 26)	23 (20.9, 25.3)
ASA				
I	343	211 (3.00%)	121 (0.86%)	11 (0.16%)
II	16,556	5,124 (72.74%)	8,605 (61.05%)	2,827 (40.79%)
III	10,749	1,683 (23.89%)	5,219 (30.02%)	3,847 (55.50%)
IV	382	25 (0.35%)	122 (0.87%)	235 (3.39%)
V	14	1 (0.01%)	2 (0.01%)	11 (0.16%)
Smoking	3,591 (12.8%)	670 (9.51%)	1,793 (12.72%)	1,128 (16.27%)
Comorbidity				
Heart disease	663 (2.36%)	116 (1.65%)	327 (2.32%)	220 (3.17%)
Hypertension	4,055 (14.46%)	896 (12.72%)	2,151 (12.26%)	1,008 (14.54%)
DM	1,573 (5.61%)	332 (4.71%)	767 (5.44%)	474 (6.84%)
Renal disease	127 (0.45%)	16 (0.23%)	57 (0.40%)	54 (0.78%)
Liver disease	785 (2.8%)	177 (2.51%)	345 (2.45%)	263 (3.79%)
Cancer	558 (1.99%)	115 (1.63%)	254 (1.80%)	189 (2.73%)
Stroke	298 (1.06%)	43 (0.61%)	134 (0.95%)	121 (1.75%)
COPD	97 (0.35%)	15 (0.21%)	39 (0.28%)	43 (0.62%)
Asthma	142 (0.51%)	35 (0.50%)	81 (0.57%)	26 (0.38%)
Thyroid disease	385 (1.37%)	97 (1.38%)	181 (1.28%)	107 (1.54%)
Preoperative Hb (g/L)	129 (117, 139)	135 (126, 145)	130 (120, 140)	119 (105, 130)
Glucose (mmol/L)	4.83 (4.38, 5.39)	4.94 (4.52, 5.44)	4.83 (4.38, 5.38)	4.69 (4.22, 5.31)
WBC (*10^9^/L)	5.78 (4.76, 7.07)	5.8 (4.84, 7)	5.71 (4.72, 6.93)	5.92 (4.76, 7.49)
Intraoperative temperature (°C)	36.4 (36.2, 36.5)	36.4 (36.2, 36.5)	36.4 (36.2, 36.5)	36.3 (36.2, 36.5)
MAP (mmHg)	80.8 (75.9, 86.1)	80.3 (75.4, 85.6)	81.1 (76.1, 86.4)	80.9 (76, 86.1)
Infusion rate (mL/kg/h)	11.5 (9.0, 14.7)	11.5 (9.0, 14.9)	11.3 (8.8, 14.4)	11.8 (9.3, 15.2)
Surgical duration	135 (95, 205)	120 (86, 181)	135 (95, 205)	156 (105, 240)
Postoperative pneumonia	898 (3.2%)	109 (1.55%)	403 (2.86%)	386 (5.57%)

Data were presented as median (interquartile) or *n* (%). ALB, albumin; BMI, body mass index; DM, diabetes mellitus; COPD, chronic obstruct pulmonary disease; Hb, hemoglobin; WBC, white blood cell; MAP, mean artery pressure.

To assess the predictive efficacy of preoperative albumin levels for postoperative pneumonia, receiver operating characteristic (ROC) curves were constructed using preoperative albumin levels (as continuous or categorized variables) along with other independent risk factors identified from multivariate regression analysis. The area under the ROC curve (AUC) was calculated using logistic regression models.

Statistical analyses were performed using Stata 16 (Stata Corp) and R software 4.12 (R Foundation for Statistical Computing). All tests were two-sided, and a *p*-value < 0.05 was considered statistically significant.

## 3 Results

Data from 22,249 patients who underwent elective major non-cardiac surgery at Chongqing University Cancer Hospital between January 2019 and December 2022 were initially available. After excluding patients with missing preoperative albumin values (*n* = 150), those with infectious diseases within 1 month before surgery (*n* = 220), and those with missing diagnostic data for pneumonia (*n* = 75), a total of 21,804 patients were included in the final analysis.

The characteristics of all patients and the three cohorts based on the first and third tertiles of preoperative albumin levels are presented in Table 1. The median age of the patients was 52 years, and the median preoperative albumin level was 42.1 g/L. Among all patients, 898 (3.2%) developed postoperative pneumonia. Patients with lower preoperative albumin levels were more likely to be smokers and to have comorbidities such as heart disease, hypertension, diabetes, stroke, renal disease, liver disease, and chronic obstructive pulmonary disease (COPD). They were also more likely to develop postoperative pneumonia.

When preoperative albumin was treated as a continuous variable in a multivariable logistic regression model, a 1 g/L increase in preoperative albumin level was associated with a 4.4% reduction in the adjusted odds ratio (aOR) (aOR: 0.956, 95% CI: 0.940–0.973, *p* < 0.001). Patients were categorized into three tertiles based on their preoperative albumin levels. Those with preoperative albumin levels <38.9 g/L or between 38.9 and 45.3 g/L exhibited a 67.4 and 30.3% higher aOR, respectively, compared to those with levels >45.3 g/L (aOR: 1.674, 95% CI: 1.313–2.135, *p* < 0.001; and aOR: 1.303, 95% CI: 1.045–1.624, *p* = 0.019) (Table 2).

**Table 2:** The association between preoperative albumin and postoperative pneumonia.

Albumin	Unadjusted OR (95%CI)	*p-*value	Adjusted OR (95%CI)	*p-*value
Continuous variable (increase 1 g/L)	0.900 (0.888, 0.912)	0.000	0.956 (0.940, 0.973)	0.000
Clinical cut-off values (g/L)				
<35	3.251 (2.732, 3.869)	0.000	1.551 (1.261, 1.907)	0.000
≥35	1.000 (reference)		1.000 (reference)	
Cut-off values (g/L) based on tertiles				
<38.9	3.752 (3.026, 4.654)	0.000	1.658 (1.301, 2.114)	0.000
38.9–45.3	1.876 (1.515, 2.323)	0.000	1.293 (1.037, 1.613)	0.022
≥45.3	1.000 (reference)		1.000 (reference)	

**Figure 1:**
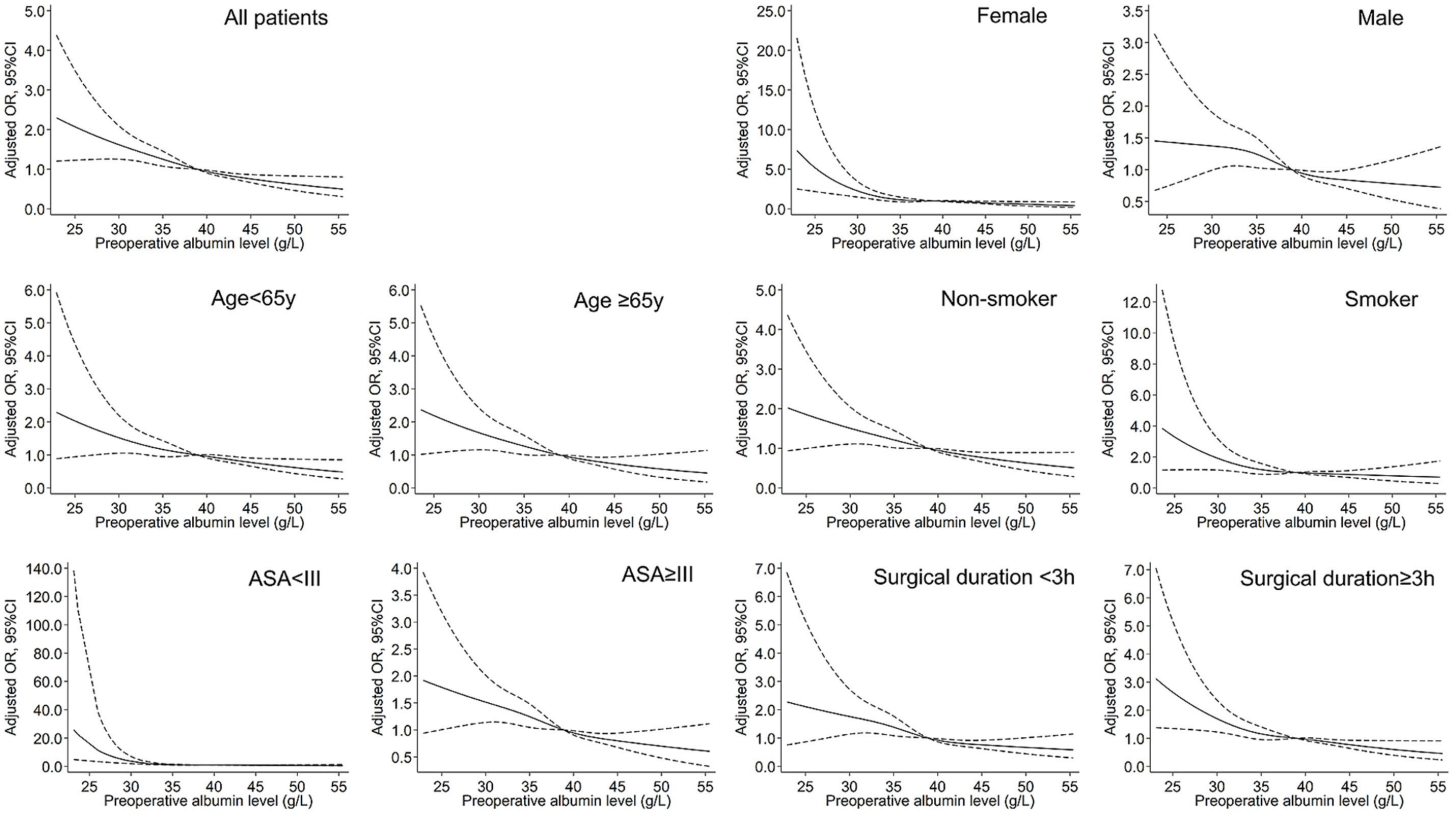
Restrictive cubic spline (RCS) models for the association between preoperative albumin and postoperative pneumonia. The solid black lines represent adjusted ORs, and the dashed lines represent upper and lower limit of 95% CI.

**Figure 2:**
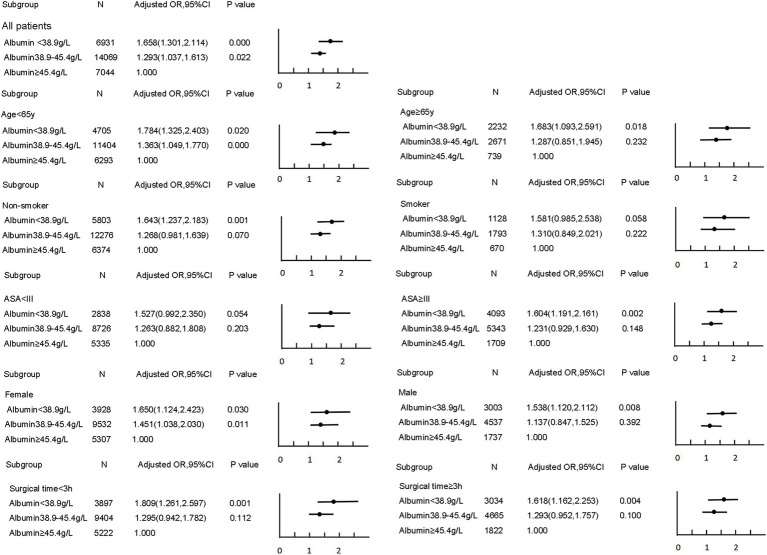
Subgroup analyses for the association between preoperative albumin and postoperative pneumonia.

A non-linear dose–response relationship between preoperative albumin levels and postoperative pneumonia was observed using a restricted cubic spline model. Similar associations were found in subgroup analyses (Figure 1). The subgroup analyses further revealed that the risk of developing pneumonia was more pronounced in younger, female, and healthier patients at lower preoperative albumin levels (Figure 2).

**Figure 3:**
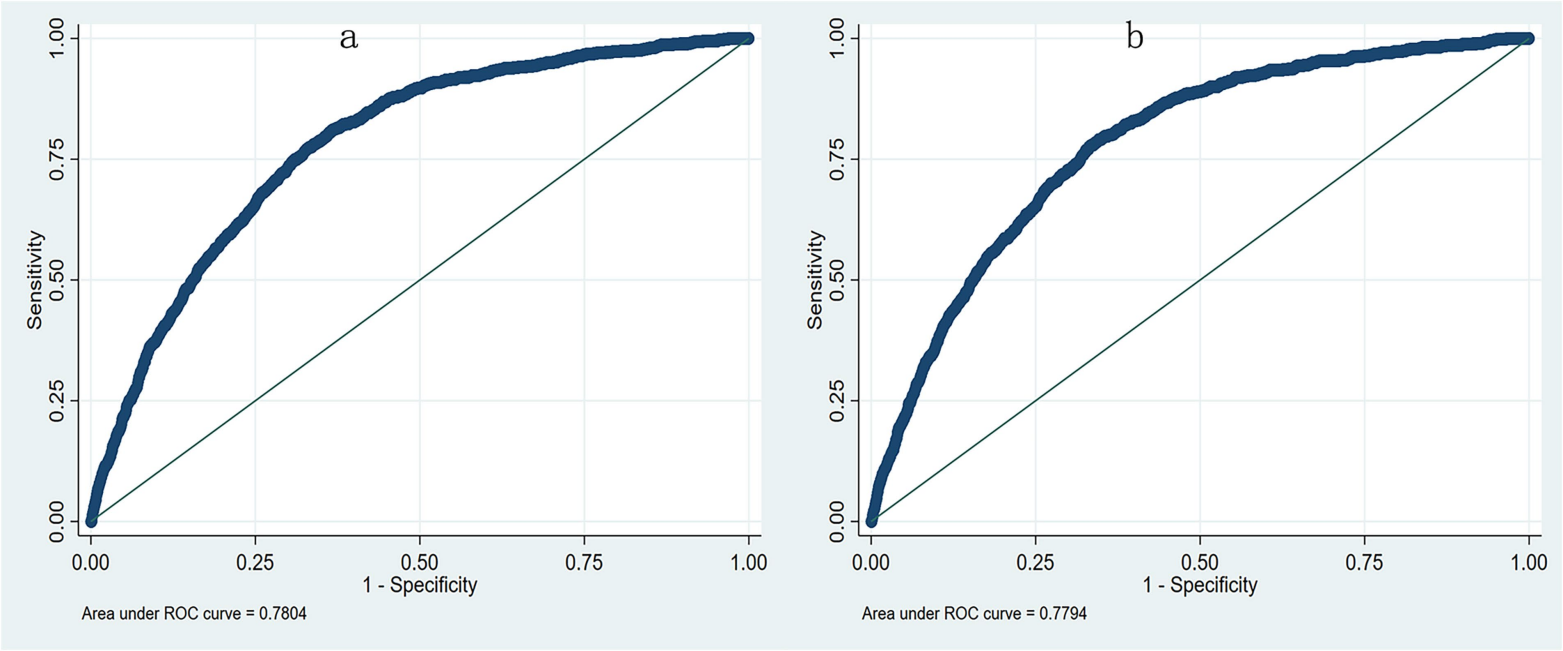
The receiver operating characteristic curve (ROC) from multivariate logistic regression model. **(a)** The AUC was 0.780 when preoperative albumin as a continuous variable; **(b)** The AUC was 0.779 when preoperative albumin as a categorized variable.

Multivariate logistic regression identified nine independent risk factors for postoperative pneumonia, including lower preoperative albumin level, older age, male, smoker, ASA > II, combined heart disease, more fluid infusion, longer surgical duration and absence of hypertension (Table 3). When preoperative albumin was incorporated as a continuous variable into the multivariable logistic regression model along with the other eight variables, the aOR was 0.960, and the AUC was 0.780. When preoperative albumin was included as a categorized variable based on the three tertiles, the AUC was 0.779 (Figure 3). Sensitivity analysis further revealed that albumin was an independent risk factor even after excluding patients with hemoglobin levels below 100 g/L (*n* = 2,369), the aOR was 0.961 (95%CI: 0.945–0.978, *p* < 0.001), and the AUC was 0.779 when considered as a continuous variable; and the aOR was 0.740(95%CI: 0.635–0.861, *p* < 0.001) when considered as a categorized variable and the AUC was 0.778.

**Table 3:** The independent risk factors from multivariate logistic regression.

Variable	OR (95%CI)	*p-*value
Age (year)	1.027 (1.020, 1.034)	0.000
Gender (male vs. female)	1.915 (1.622, 2.261)	0.000
ASA (III ~ V vs. I ~ II)	2.019 (1.765, 2.521)	0.000
Smoker (presence vs. absence)	1.561 (1.317, 1.850)	0.000
Heart disease (presence vs. absence)	1.460 (1.077, 1.978)	0.015
Hypertension (presence vs. absence)	0.801 (0.662, 0.970)	0.023
Preoperative albumin (g/L)	0.960 (0.946, 0.974)	0.000
Fluid infusion rate (mL/kg/h)	1.018 (1.002, 1.034)	0.026
Surgical duration (min)	1.002 (1.002, 1.003)	0.000

OR, odds ratio; 95%CI, 95% confidence intervals; BMI, body mass index; DM, diabetes mellitus; WBC, white blood cell.

## 4 Discussion

This retrospective cohort study demonstrated that preoperative albumin levels were associated with postoperative in-hospital pneumonia in a non-linear response manner in patients undergoing major non-cardiac surgeries. The cut-off points in our study were based on tertiles of preoperative albumin levels (<38.9 g/L, 38.9–45.3 g/L, and >45.3 g/L). Compared with the highest tertile, *n* albumin level between 38.9 and 45.3 g/L was associated with a 30% increase in risk, and a level <38.9 g/L was associated with a 67% higher risk of postoperative pneumonia.

Preoperative hypoalbuminemia has been associated with postoperative adverse outcomes (9, 12, 13). For instance, Marin et al. (14) analyzed postoperative outcomes in 100 patients undergoing minor esophageal or gastric surgeries and found that patients with normal albumin levels (>35 g/L) had lower mortality. Kudsk et al. (15) conducted a retrospective study of 706 patients undergoing foregut surgery and reported that those with lower preoperative serum albumin levels had a higher incidence of postoperative complications and mortality. Wang et al. (7) identified preoperative hypoalbuminemia as a predictor of postoperative pneumonia through multivariable regression analysis in femoral neck fracture surgery. In a large retrospective study involving 135,008 patients undergoing total joint arthroplasty, preoperative hypoalbuminemia (<35 g/L) was an independent risk factor for postoperative adverse outcomes, including infection, pneumonia, sepsis, and myocardial infarction (9). Bendersky et al. (16) reported that the optimal threshold for preoperative albumin levels to predict postoperative complications in colorectal surgery was 39 g/L. In our study, a preoperative albumin level <45.3 g/L was predictive of postoperative pneumonia, which is higher than the threshold reported by Bendersky.

Malnutrition, often linked with elderly patients, female sex, and comorbidities (17, 18). In our study, patients with lower albumin levels were more likely to have comorbidities such as anemia, heart disease, hypertension, diabetes, stroke, renal disease, liver disease, and COPD. Serum albumin, the most abundant plasma protein, can be influenced by factors such as nutrition, liver dysfunction, malignancy, stress, and comorbidities (e.g., diabetes) (19), and its level serves as an indicator of nutritional status. Low albumin levels can impair immune function, increasing the likelihood of infectious complications after surgery (20).

Our results suggest that the optimal preoperative albumin level (45.3 g/L) for predicting postoperative pneumonia is higher than the clinical cutoff criteria for hypoalbuminemia (<35 g/L). While this statistically derived threshold is higher than the conventional 35 g/L diagnostic level, we suggest it not serve as a strict contraindication to surgery, but as a trigger for preoperative nutritional evaluation. In practice, levels below 45.3 g/L should prompt further assessment using tools such as NRS-2002 and consideration of time-limited nutritional interventions when clinically appropriate.

Interestingly, at lower preoperative albumin levels, the risk of pneumonia was more pronounced in younger, female, and generally healthier patients (ASA I-II). Elevated estrogen levels in young women may affect lung epithelial barrier integrity through immunomodulatory pathways. Under hypoalbuminic conditions, this regulation can become disrupted, leading to impaired clearance of lung pathogens (21). Moreover, hypoproteinemia, serving as a stress indicator, may indicate subclinical inflammation or metabolic strain. Unlike chronically ill patients, who may exhibit adapted immune response, healthier patients could experience exaggerated inflammatory reactions following surgical stress and hypoalbuminemia, thereby increasing pneumonia risk (22). Consequently, elective surgery might be deferred until an optimal preoperative albumin level is attained, thereby mitigating the risk of postoperative pneumonia and other potential complications.

Our data analysis further identified surgical duration as an independent risk factor for postoperative pneumonia. Notably, patients in the lower albumin group experienced prolonged surgical durations, which contributed to extended periods of general anesthesia and intraoperative mechanical ventilation. Longer procedures are associated with greater surgical trauma and physiological stress, often necessitating increased administration of opioids, sedatives, and neuromuscular blocking agents. These factors collectively impair respiratory function, ciliary clearance, and immune response, thereby elevating the risk of pulmonary complications. Furthermore, extended operative time may lead to a higher likelihood of prolonged postoperative mechanical ventilation and longer ICU stays, both of which are established risks for developing postoperative hospital-acquirement pneumonia (23, 24). However, our study lacks detailed perioperative data related to medication dosing, ventilation parameters, or precise ICU course. Therefore, residual confounding or unmeasured cross-effects between nutritional status and operative factors cannot be ignored. Further studies incorporating these data are warranted to disentangle these complex interactions.

Several limitations should be acknowledged. First, only in-hospital pneumonia was recorded, thus excluding later-onset cases. Second, this is a retrospective, single-center study, and the generalizability of the results is limited. Third, we did not adjust the risk ratio for certain immunosuppressive conditions such as HIV, chronic wasting diseases, or chemotherapy history (25). Future studies should incorporate these biomarkers of immune function. Additionally, perioperative nutritional support such as albumin infusion or enteral nutrition was not documented, potentially introducing unmeasured confounding. Large-scale, randomized controlled trials are warranted to validate these observations.

In conclusion, preoperative albumin level is significantly associated with postoperative pneumonia in major non-cardiac surgery patients. A threshold of 45.3 g/L appears more predictive than the conventional 35 g/L cutoff. Further research is needed to determine whether preoperative nutritional interventions aim at raising albumin can reduce the incidence of pneumonia and other postoperative complications.

## Data Availability

The raw data supporting the conclusions of this article will be made available by the authors, without undue reservation.
